# Risk factors and complications of small for gestational age

**DOI:** 10.12669/pjms.35.5.253

**Published:** 2019

**Authors:** Qiang Liu, Hui Yang, Xuemei Sun, Guimei Li

**Affiliations:** 1 Dr. Qiang Liu, Department of Newborn, Linyi People’s Hospital, Linyi, Shandong, 276003, China; 2 Dr. Hui Yang, Department of Anesthesiology, Linyi People’s Hospital, Linyi, Shandong, 276003, China; 3 Dr. Xuemei Sun, Department of Newborn, Linyi People’s Hospital, Linyi, Shandong, 276003, China; 4 Dr. Guimei Li, Department of Pediatrics, Shandong Provincial Hospital Affiliated to Shandong University, Shandong University, Jinan, Shandong, 250021, China

**Keywords:** Complications, Risk factors, Small for gestational age

## Abstract

**Objective:**

The objective of this study was to find out the maternal risk factors and perinatal complications in small for gestational age (SGA) newborns.

**Methods:**

A total of 181 SGA cases and 1299 cases of appropriate for gestational age (AGA) with the same gestational age between January 2015 and December 2016 were enrolled in Linyi People’s Hospital, China. The risk factors were analyzed and the frequencies of perinatal complications were compared between the two groups.

**Results:**

The frequencies of maternal risk factors such as pregnancy-induced hypertension, abnormal placenta and twins in the SGA group were significantly higher than that in the AGA group (P<0.05). The incidence of hyperbilirubinemia and hypoglycemia in the perinatal period was also higher in the SGA newborns group (P<0.05), while there were no significant differences in the incidence of pneumonia, apnea, septicemia, intracranial hemorrhage, neonatal asphyxia, congenital malformations, hypoxic-ischemic encephalopathy, respiratory distress syndrome and necrotizing enterocolitis between the two groups.

**Conclusions:**

SGA can cause perinatal complications including neonatal hypoglycemia and hyperbilirubinemia. It is necessary to strengthen the perinatal monitoring and antenatal care to reduce SGA and the perinatal complications of SGA.

## INTRODUCTION

Small for gestational age infant (SGA) was defined by the WHO in 1995 as the infant with birth weight below the 10th percentile of mean body weight of the infants of same gestational age and same gender.^1^ In 2001, the World SGA Development Conference announced that SGA refers to birth weight and/or body length below averagely two standard deviations (≤-2s) of same gestational age, about the third percentile of body weight and/or body length of same gestational age.^2^

SGA has higher mortality than infants with normal birth weight, and is one of the major causes of perinatal death.^3^,^4^ SGA not only threatens the growth and development of fetus in the uterus, but also affects physical and mental development in childhood and adolescence,^5^,^6^ causing learning difficulties, language barriers and cognitive and behavioral defects^7^-^9^, and even increasing the incidence of autism.^10^ SGA is closely related to some important diseases in adulthood.^11^ The prevalence of SGA births has been reported to be 8.6%–11.4% in several countries,^12^ while in China, the overall prevalence of SGA is estimated to be 8%-13%.^13^

Premature infants, term infants and post term infants may develop SGA. The causes are complex, and many studies always aimed at a single risk factor for research, such as mother’s age, socioeconomic status and education, body mass index and obesity, prenatal diet and nutrition, diseases in pregnancy, smoking, placental conditions, etc. Pregnancy-induced hypertension is one of the main causes of SGA.^14^ It is a common complication in pregnancy and is the leading cause of maternal and perinatal fetal death. Pregnancy-induced hypertension affects fetal development in the uterus by impairing uterine artery, myometrial spiral artery, basilar artery, placental blood vessels and umbilical artery.^15^ It should be noted that elderly pregnant women are prone to pregnancy-induced hypertension and other complications, leading to occurrence of SGA.^16^ SGA infants have many perinatal complications, commonly preterm birth NEC (necrotizing enterocolitis) and adverse perinatal outcome, and is the second major cause for perinatal diseases and death, following premature birth, but there are still differences between the research findings, such as hypoglycemia.^17^,^18^ In order to find out the risk factors and complications of SGA, SGA and AGA cases were retrospectively collected from a tertiary-care hospital in China and analyzed in this study.

## METHODS

This was a retrospective case-control study, which was approved by the Ethics Committee of Linyi People’s Hospital. A total of 181 SGA and 1299 AGA cases (controls) from January 2015 to December 2016 were enrolled in Linyi People’s Hospital, China. Sampling was not applied. The SGA cases accounted for 12.23% (181/1480) of all subjects, consistent with the overall prevalence of China (8%-13%). The sample size of SGA was consistent with previous studies.^19^ The diagnostic criteria for SGA referred to the neonatal normal weight standard of different gestational ages,^20^ as below the 10th percentile of birth weight of the overall population of same gestational age.

### Clinical information

Information including pregnant mother’s general demographic characteristics, perinatal complications including neonatal asphyxia, congenital malformations, hypoglycemia, neonatal hypoxic-ischemic encephalopathy, neonatal respiratory distress syndrome, neonatal necrotizing enterocolitis, neonatal pneumonia, neonatal hyperbilirubinemia, neonatal apnea, neonatal sepsis and neonatal intracranial hemorrhage, and physiological indicators including gestational age, birth weight and gender was collected by reviewing the electronic neonatal and obstetric records with complete information.

### Statistical analysis

Patients’ characteristics were summarized by mean ± SD for continuous variables and frequency for categorical variables. Independent two-sample t-test and chi-square test were used for group comparisons. Normal distribution assumptions were examined using Q-Q plots. SGA risk factors were identified by logistic regression. The risk factors in univariate analysis (p-value <0.1) were included. Gestational age and gender were also included. The odds ratio of SGA for each factor was calculated. Logistic regression was also used to evaluate SGA as a risk factor for different perinatal complications. The predictive abilities of models were reasonable with C-statistics presented. All statistical tests were conducted at a two-sided 5% significance level using SPSS 21.0.

## RESULTS

SGA occurred more frequently at low gestational age (P<0.001), low birth weight (P<0.001), gender (female) (P<0.001), twins (P=0.002), and the differences were statistically significant (P<0.001). Compared with the control group, the mothers of the SGA group were more likely to have placental abnormalities in pregnancy (P=0.044) and PIH (P<0.001). The frequencies of hypoglycemia (P<0.001) and of hyperbilirubinemia (P<0.001) were significantly higher in the SGA group than in the control group, while there were no significant differences in the incidence of other examined complications. Table-I

**Table I:** Basic description.

	Cases(n=181)	Controls (n=1299)	P-value*
Gestational Age(weeks)	36.24 (2.252)	36.6 (3.170)	<0.001
Birth weight(g)	1926.35 (590.538)	2875.67 (793.309)	<0.001
Male, *n*	85(46.96%)	823(63.36%)	<0.001
***Risk Factors***			
Twins, *n*	33 (18.23%)	135 (10.39%)	0.002
Premature rupture of membrane, *n*	16 (8.84%)	174 (13.39%)	0.086
Abnormality of amniotic fluid, *n*	57 (31.49%)	370 (28.48%)	0.403
Abnormality of umbilical cord, *n*	217 (14.92%)	1082 (16.71%)	0.544
Abnormality of placenta, *n*	25 (13.81%)	118 (9.08%)	0.044
Abnormal labor, *n*	20 (11.05%)	170 (13.09%)	0.443
Pregnancy-induced hypertension syndrome, *n*	38 (20.99%)	76 (5.85%)	<0.001
Intrahepatic cholestasis of pregnancy, *n*	16 (8.84%)	95 (7.31%)	0.465
Anemia, *n*	9 (4.97%)	51 (3.93%)	0.504
Smoking or Passive Smoking, *n*	23 (12.71%)	151 (11.62%)	0.672
Advanced paternal age, *n*	7 (3.87%)	71 (5.47%)	0.367
***Perinatal complication***			
Asphyxia, *n*	41 (22.65%)	339 (26.10%)	0.320
Congenital malformation, *n*	26 (14.36%)	162 (12.47%)	0.474
Hypoglycemia, *n*	39 (21.55%)	148 (11.39%)	<0.001
Hypoxie-ischemic encephalopathy, *n*	46 (25.41%)	381 (29.33%)	0.276
Neonatal respiratory distress syndrome, *n*	29 (16.02%)	243 (18.71%)	0.382
Neonatal necrotizing enterocolitis, *n*	8 (4.42%)	39 (3.00%)	0.308
Pneumonia, *n*	69 (38.12%)	528 (40.65%)	0.516
Hyperbilirubinemia, *n*	66 (36.34%)	316 (24.31%)	0.001
Apnea, *n*	5 (2.76%)	41 (3.16%)	0.775
Sepsis, *n*	5 (2.76%)	59 (4.54%)	0.270
Intracranial Haemorrhage, *n*	23 (12.71%)	225 (17.32%)	0.119

* P-value was generated from t-test or Pearson chi-square test.

Gender (p-value<0.001), Twins (p-value=0.004) and Pregnancy-induced hypertension (p-value < 0.001)) were the risk factors of SGA. Table-II. Logistic regression model found that SGA was risk factor of perinatal complications: hypoglycemia (p-value <0.001) and hyperbilirubinemia (p-value <0.001). Table-III

**Table II:** Risk factors for SGA using logistic regression model with multiple covariates.

	OR	95% CI	P
Gestational Age (weeks)	0.978	(0.923,1.036)	0.444
Gender (Male)	0.537	(0.390,0.741)	<0.001
Twins	1.946	(1.233,3.073)	0.004
Premature rupture of Membrane	0.624	(0.356,1.093)	0.099
Abnormality of placenta	1.626	(0.997,2.653)	0.051
Pregnancy-induced -Hypertension syndrome	3.923	(2.533,6.077)	<0.001
C-statistic= 0.698.			

**Table III:** Effect of SGA on the Perinatal Complications.

	Neonatal hypoglycemia		Neonatal Hyperbilirubinemia	

	OR (95%CI)	P	OR (95%CI)	P
SGA	2.081 (1.393,3.111)	<0.001	0.530 (0.368,0.763)	0.001
Gestational -Age	0.853 (0.812,0.896)	<0.001	0.871 (0.840,0.903)	<0.001
Gender (Male)	0.844 (0.612,1.163)	0.299	0.996 (0.794,1.250)	0.975

Each column represents one logistic model with the perinatal complication as the outcome. The C-statistic for each model are 0.677, 0.605, respectively.

## DISCUSSION

The causes and pathogenesis of SGA remain unclear and have not previously been well characterised. This study analyzed the risk factors and complications of SGA with data collected from a tertiary-care hospital in China.

Although the identified risk factors of SGA varied among studies, it is mostly believed that the risk factors include obstetric and fetal factors. This study showed that pregnancy-induced hypertension of pregnant mother, twins, female infant are common causes of SGA. Consistent with previous reports,^14^ pregnancy-induced hypertension remained to be a major cause of SGA, which is known to affect fetal development in the uterus. Meanwhile, the multiple birth-caused SGA were likely due to the unbalanced placental blood supply or inter-fetal blood transmission, which further lead to insufficient oxygen or nutrient supply. Unlike previous observation^21^, in this study it was found that female infant was a cause of SGA and thus further validation is needed. Studies have found that pregnancy with severe anemia^22^, smoking or passive smoking of mothers^23^,^24^ are risk factors of SGA, which however were not confirmed in this study. That might be due to the difference in the studied population as it has been reported that ethnicity was a factor associated with SGA,^25^ and in this study, all subjects were Chinese.

It is known that SGA newborns have high rates of morbidity and mortality and may also suffer from malnutrition and life-long complications. In addition, SGA often causes a variety of perinatal complications.^26^ In this study, it was found that hypoglycemia and hyperbilirubinemia were perinatal complications of SGA. SGA newborns might have a condition with low hepatic glycogen storage, high glucose consuming due to disease stress, and/or unstable glucose metabolism that could result in hypoglycemia. Therefore, although the hypoglycemia could improve along with growth, it is important to conduct close monitoring of blood glucose level for the newborns to avoid hypoglycemia-induced impairments. The SGA fetus might embedded in a relatively anoxic environment, and the consequent conditions such as compensatory increase of the red blood cell number and/or acidosis might lead to hyperbilirubinemia.^27^,^28^

## CONCLUSION

In conclusion, SGA is the result of multi-factor interaction and it can cause perinatal complications including neonatal hypoglycemia and hyperbilirubinemia.

### Author`s Contribution

**GL** conceived, designed and did statistical analysis & editing of manuscript.

**QL, HY and XF** did data collection and manuscript writing.

**QL and GL** did review and final approval of manuscript.
